# Inequality of access to advanced therapies for patients with inflammatory arthritis: a postcode lottery?

**DOI:** 10.1093/rap/rkab081

**Published:** 2021-11-10

**Authors:** Arvind Kaul, Jatin Mistry, Annamaria Iagnocco, Xenofon Baraliakos, Ailsa Bosworth, Iain McNicol

**Affiliations:** 1 Institute of Medical and Biomedical Education, St. George’s University of London; 2 Department of Rheumatology, St. George’s University Hospitals NHS Foundation Trust, London, UK; 3 Academic Rheumatology Centre, Università Degli Studi Di Torino, Torino, Italy; 4 Rheumazentrum Ruhrgebiet Herne, Ruhr-University, Bochum, Germany; 5 National Rheumatoid Arthritis Society, Maidenhead, Berkshire, UK

**Keywords:** advanced therapies, access, inequality, RA, PsA, axial spondyloarthritis

## Abstract

**Objectives:**

Advanced therapies (AT), including biologics, biosimilars and Janus kinase inhibitors, have dramatically improved the quality of life of patients with RA, PsA and axial spondyloarthritis (axSpA). Evidence-based criteria for prescribing these drugs in England and Wales is formulated by the National Institute for Health and Care Excellence (NICE) through health technology appraisals and guidelines, with the aim of providing equitable access to AT for patients with severe or resistant disease. Similar bodies exist in some, but not all European countries, with disparities in AT access between countries for RA. We examined whether this disparity was mirrored in England for RA, PsA and axSpA despite the National Health Service in England and Wales being legally obliged to provide funding for AT recommended by NICE’s Health Technology Appraisal board, through the commissioning bodies, the clinical commissioning groups (CCGs).

**Methods:**

We requested AT pathways from CCGs in England. Where these were not available, individual hospital Trusts were contacted using freedom of information requests.

**Results:**

We found marked variability in the way that CCGs in England interpret NICE guidance. We found 41, 29 and 25 different pathways for RA, PsA and axSpA, respectively. Similar disparities existed with sequential prescribing where one AT did not work, with limits on the numbers of sequential AT in 54%, 59% and 59% of CCGs for RA, PsA and axSpA, respectively, and with these limits being different for the same condition between CCGs.

**Conclusion:**

Although patients at identical stages of their disease course should have access to the same NICE-approved AT, we found this is not the case for large parts of England. Inequality of access was found between regions, mirroring the variability that occurs between countries throughout Europe. Harmonization of access needs to be addressed by policymakers to ensure fairness in the way that clinicians and patients can access AT.

Key messages

- National Institute for Health and Care Excellence guidance for inflammatory arthritis should provide equal access to advanced therapies.
- National Institute for Health and Care Excellence guidance is implemented in England by clinical commissioning groups; we demonstrate pathway variations by geographical region.
- This postcode lottery in access to advanced therapy restricts optimal management based on cost and geography.

## Introduction

Advanced therapies (AT; biologics, biosimilars and Janus kinase inhibitors) have revolutionized the management of RA, PsA and axial spondyloarthritis (axSpA). AT have a significant financial impact on health care. In 2020, global sales of Humira exceeded US$19 billion [1].

Locally based NHS decisions previously led to inequitable funding provision for therapies in England and Wales. This situation led to the development of harmonized evidence-based standards by the National Institute for Health and Care Excellence (NICE) through health technology appraisals and guidelines, permitting in Rheumatology, equitable access to AT on meeting criteria. The National Health Service (NHS) in England and Wales is legally obliged to fund therapies recommended by the NICE health technology appraisals board. Similar AT approval bodies exist in Europe (e.g. The Institute for Quality and Efficiency in Health Care in Germany), but AT funding processes vary between countries for RA [2].

Responsibility for funding AT in England is delegated to clinical commissioning groups (CCGs). We examined whether pathway variability between CCGs promotes inequality of access to AT within England by examining pathway concordance with the NICE guidance principles of equitable access.

## Methods

All 135 CCGs in England (post CCG merger from 1 April 2020) were sent freedom of information requests for their AT pathways for RA, PsA and axSpA. We estimate that data covered most of the >392 000 RA patients in England [3], 100 000 PsA patients [4] and similar numbers with axSpA [5]. We requested information on pathways for AT and the maximum number of AT commissioned for these conditions before an individual funding request was required. Individual funding requests allow consideration of funding for health-care interventions outside the treatments that CCGs have agreed to fund.

Some CCGs directed us to contact acute Trusts for information. Responses were recorded under the CCG for that Trust. All CCG pathways were current as of May 2020 and were compared with the relevant NICE guidance [6–14].

The AT pathways were given a unique number so that common CCG pathways were identifiable. The responses of some CCGs were unclear, no information was sent, or a review of pathways was underway.

Numbers of commissioned AT were recorded. The CCG ‘local pathways’ were analysed to determine whether numbers of AT commissioned were stated. If not, or if the CCG did not publish local pathways, we used responses to freedom of information requests for clarification on whether there was a local AT pathway for RA, PsA and AS and maximum ATs the CCG would commission. Using this method, we did not find any situations in which freedom of information and local pathway information differed. Email confirmation was sought when no pathway existed. Where a clear answer was not provided, ‘N/A’ (not available) was recorded, and where the CCG responded that there was no limit, restriction or hierarchy regarding the number of biologics a patient could attempt, ‘No Cap’ was recorded. No ethical approval was required for this study.

## Results

**Fig. 1:**
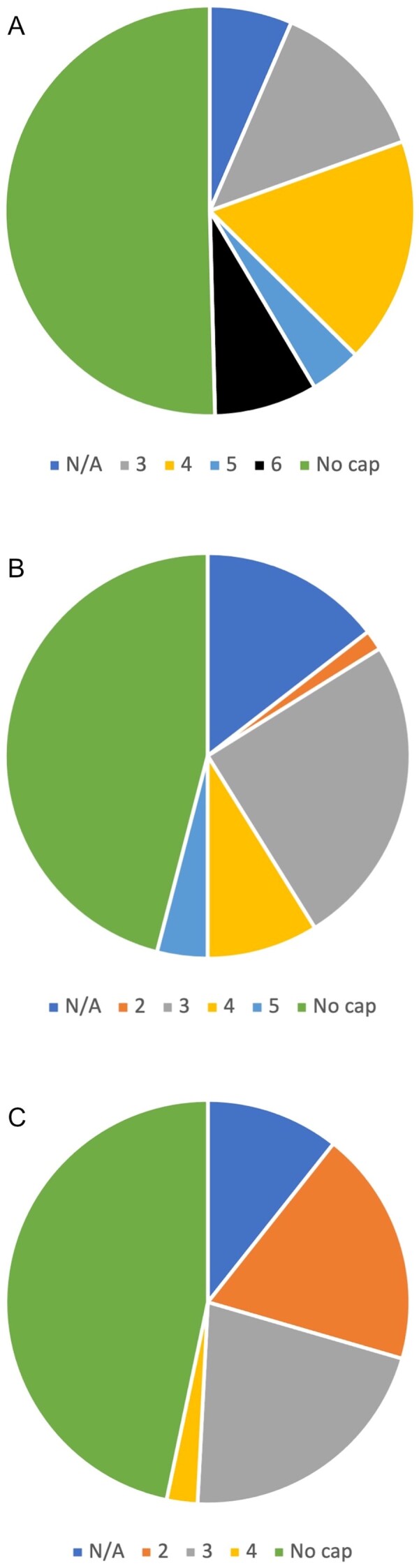
Variation in sequential prescribing limits in RA (**A**), PsA (**B**) and axial spondyloarthritis (**C**) N/A: data not available.

Clinical commissioning group responses were obtained from 123 of 135 CCGs for RA, 122 of 135 for PsA and 119 of 135 for axSpA (Table 1; Fig. 1). Overall, we found 41 distinct pathways for RA, 29 for PsA and 25 for axSpA, demonstrating regional variation and variable interpretation of NICE guidance. All pathways allowed anti-TNF-α as first-line therapy. There was no specified pathway in 44 of 123 (35%) responses for RA, 59 of 120 responses for PsA (49%) and 62 of 119 responses for axSpA (52%). Where there was no specified pathway, the responses confirmed by the CCGs or Trusts was that they had no specific order of AT, allowing clinicians to prescribe any AT in any order without any hierarchy, provided NICE guidance was followed.

**Table 1:** Details of the clinical commissioning group advanced therapy prescribing pathway for each indication

	RA	PsA	AxSpA
CCGs contacted, *n*	135	135	135
CCG responses received, *n*	123	120	122
No or unclear response, *n*	12	15	13
Distinct pathways, *n*	41	29	25
No specified pathway, *n* of CCGs	44	59	62
Sequential prescribing limits			
No cap	53	57	57
2 AT	0	2	23
3 AT	15	31	26
4 AT	22	11	3
5 AT	6	5	0
6 AT	10	0	0

Sequential prescribing limits are given for interpretable responses only. These included similar or different modes of action as first and subsequent lines. The CCGs could also have ‘no specified pathway’ with either ‘no cap’ or a variable cap. AT: advanced therapies; axSpA: axial spondyloarthritis; CCG: clinical commissioning group.

Where distinct pathways existed, the rationale in directing the choice of AT for the formulation of the pathway was sought (see Supplementary Fig. S1, available at *Rheumatology Advances in Practice* online, for RA). The commonest rationale for distinct pathways was cost (8 of 41, 19%) with the commonest combination reason being cost, clinical factors and patient choice (17 of 41, 41%). In 3 of 41 (7%), no rationale was provided (Supplementary Fig. S1, available at *Rheumatology Advances in Practice* online).

Where pathway information was recorded, we determined limits (cap) on the number of and/or the lines of NICE-approved AT that could be prescribed before an individual funding request was needed. Surprisingly, for NICE-approved AT, supposedly all legally available to prescribe on fulfillment of criteria, limits varied by region, including limits for specific drugs. Some pathways specified etanercept biosimilar first line, followed by other anti-TNF-α second/third line, whereas other pathways approved different mode of action (MoA) as second/third line.

Where data were interpretable (113, 113 and 111 CCGs for RA, PsA and axSpA, respectively), we found that 46, 41 and 41% of CCGs did not specify a cap on the number of AT that could be prescribed for RA, PsA and axSpA, respectively. However, this meant that limits were placed in 54, 59 and 59% of the interpretable CCG pathways for the respective conditions. Limits varied between CCGs, with some permitting two lines of RA therapy, others more, despite five different NICE-approved AT MoA. Anomalies existed where four lines of AT were permitted for axSpA despite only two NICE-approved MoA (anti-TNF-α and secukinumab) because three different anti-TNF-α were approved in series followed by secukinumab.

## Discussion

We found inconsistency in the interpretation of AT NICE guidance for RA, PsA and axSpA in England by CCGs. Identical patients could be subject to different prescribing arrangements depending on location. NICE was designed to ensure equal access to therapies regardless of geography. Variations in access to AT for RA also exist in comparable European bodies despite similar stated aims [2].

The number of CCGs with specified pathways was highest for RA, where several AT MoA are available. This suggests greater AT choice is counterbalanced by more restrictive pathways. NICE Multiple Technology Appraisal covering several MoA states that the cheapest drug should be used, but not sequential biologic order (an exception, rituximab for RA, is only allowed second line, unless anti-TNF-α is contraindicated). We found many CCG pathways mandated sequential AT, specified first line and the order of subsequent AT. After first line, usually anti-TNF-α, second-line agents varied by region for the same indication. Of concern, some pathways did not permit a return to previously used MoA, irrespective of secondary rather than primary failure.

Clinical commissioning groups took cost as the commonest arbiter of AT choice, explicitly stated in some pathways. However, phrasing of NICE guidance encourages disparity. NICE states that ‘If more than one treatment is suitable the least expensive (taking into account administration costs and patient access schemes) should be chosen’. However, ‘If more than one treatment is suitable’ could mean a similar MoA (e.g. different anti-TNF-α therapies) or different MoA (e.g. anti-IL-6, Janus kinase inhibitors). Thus, although anti-TNF-α drugs might be equally efficacious for RA (similar MoA), etanercept biosimilar is cheaper than golimumab bio-originator. Patients, for reasons of travel or convenience, might prefer four-weekly golimumab to weekly etanercept. We found that this type of variation was not permitted in some pathways despite NICE guidance in some health technology appraisals stating ‘consider patient choice’.

Commissioning arrangements cause inequality in other rheumatic conditions. Between 2015 and 2017, NHS England commissioning for bosentan and digital ulcers in scleroderma was introduced, with no similar arrangements in Wales. Consequently, bosentan prescriptions in England increased by 47%, but were stable in Wales, where individual funding requests were needed [15].

Although all devolved UK nations use NICE guidance to direct AT eligibility, access varies by nation (see Acknowledgements). The Scottish Medicines Consortium approves AT for local health boards if clinicians wish to prescribe, although NICE AT have no formal status. (E. Murphy and A. Macdonald, personal communication). Wales follows NICE guidance through four Health Boards. If NICE criteria are fulfilled, no AT hierarchy exists. In Northern Ireland, the five health-care Trusts follow NICE guidance. A managed entry programme mandates biosimilars as first line, but subsequent AT can be prescribed in any order if NICE approved. These arrangements might change in future owing to cost, with more pathway-orientated prescribing, similar to England (R. Lennon, personal communication). When a clinician requests an AT that falls outside the CCG sequencing, an individual funding request is needed despite AT being legally permitted on fulfilment of NICE criteria in England. Individual funding request processes, in our experience, are time consuming and opaque and can be approved for one patient but not another in identical circumstances. Individual funding requests in Northern Ireland are reserved for AT that are licensed but not NICE approved rather than those that are, as in England.

Wide disparities in access arrangements for RA AT occur in European countries despite similar pledges by public funding bodies. From a survey of 46 European countries [2], 10 did not reimburse any AT. Requirements for RA disease duration varied in 34% of countries, and 64% had no requirement. The required DAS varied, with 56% having DAS28 > 3.2 as a cut-off, but many countries, such as Switzerland, Ireland and Luxembourg, had no requirement. Using a population model and EULAR recommendations, Kaló *et al*. [16] estimated that 32% of the RA population in Europe were eligible for AT, but only 18% were prescribed AT. As a result, 700 000 RA patients would be excluded from AT treatment, because national eligibility and reimbursement criteria are discordant with EULAR recommended eligibility criteria.

Variations in health-care systems are likely to contribute to inter-European disparities. Article 32 of the Italian constitution protects health as a fundamental right of the individual. The Italian Medicines Agency grants ‘guaranteed access to medicines and their safe and appropriate use as a health protection instrument’. However, Italy’s public health system has 21 autonomous areas, with each region having distinct AT access arrangements and with consequent lack of consistency. As with England, cost is frequently a primary determinant in providing access to AT, rather than patient or clinician choice (M. Galeazzi, personal communication).

In Germany, through the Institute for Quality and Efficiency in Health Care, AT access is free for all patients with RA, axSpA or PsA. Physicians initiate or switch AT provided the reason is documented. Biosimilars are preferred to bio-originators, and quotas (different for each region) for the proportion of biosimilars used, need to be fulfilled (X. Baraliakos, personal communication). In Belgium, clinicians have freedom to prescribe whichever biologic MoA they feel is best for the patient at any stage of treatment, with no hierarchy (R. Lories, personal communication).

Variations in access are not only caused by commissioning arrangements. In Canada, females, patients in urban areas and younger patients had their first AT sooner despite identical health-care coverage. Variations in the practice of health-care professionals might determine these variations in access [17]. Across Europe, deprivation, geography, clinical practice, demand and finance all affect access to health care, whereas guidelines may standardize practice and reduce variations [18]. Levesque *et al*. [19] suggested five domains affecting accessibility to interventions: approachability (when patients recognize there is a service available), acceptability (acceptance of the cultural and social implications of therapy), availability (health-care interventions can be reached in a timely manner), affordability (people or services having the economic capacity to provide or engage with the intervention) and appropriateness (the fit between intervention, service and the patient’s needs). Any or all of these factors may be relevant to AT access.

The strengths of our study include comprehensive responses from the majority of CCGs covering most of the RA, PsA and axSpA patients in England. This is the first study, to our knowlege, to show variable AT access in conditions other than RA and to demonstrate that access problems are generic across conditions. The weaknesses of the study include data collection from one country only. Larger studies to collect similar data across Europe might show broader geographical access variations, to complement known disparities across Europe [2]. Data collection from less wealthy nations might confirm that AT access is related to socioeconomic wealth [2], a factor that affects numerous health-care interventions [18].

In May 2020, NHS England’s Regional Medicines Optimization Committee stated that commissioning policies which limit patients’ access to appropriate treatments based on prior treatment numbers go against the NHS Constitution. Clinical assessment of the appropriateness of treatment should override policy implementation for cost saving [20].

This interpretation of NICE guidance harmonizes treatment access in the way NICE intended. We suggest that clear guidance from regulatory authorities on AT prescribing is essential to uphold this principle. Work is needed to realize these principles within the UK and Europe, where equality is embedded in constitutional arrangements. Clinical, political and financial discussions are needed to ensure that reimbursement criteria for AT are translated into equity of access for patients with RA, PsA and axSpA.

## Supplementary Material

rkab081_Supplementary_Data
