# Supplementary material for: Inequality of access to advanced therapies for patients with inflammatory arthritis: a postcode lottery?
Source: Rheumatol Adv Pract. 2021 Nov 10;5(3):rkab081. doi: 10.1093/rap/rkab081 (PMC8634385; doi:10.1093/rap/rkab081)
Supplement: rkab081_Supplementary_Data [file rkab081_supplementary_data.docx]

Supplementary Figure S1. Numerical reasons given by CCGs to justify the order of ATs in 41 rheumatoid arthritis pathways


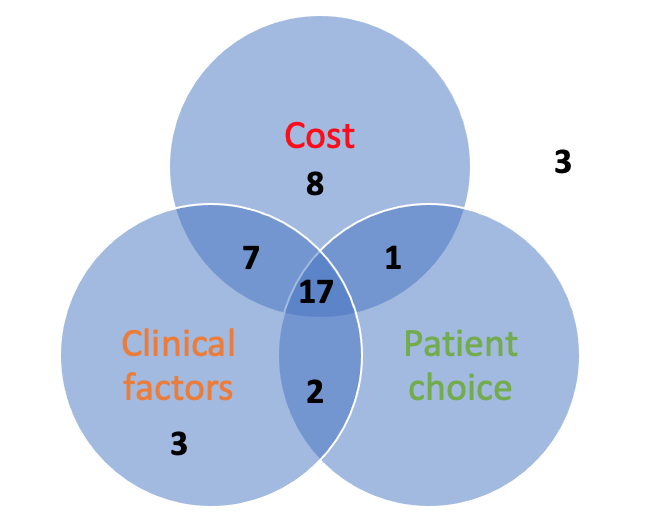


No reason
